# Supplementary material for: Gut Microbiome Profiles and Associated Metabolic Pathways in HIV-Infected Treatment-Naïve Patients
Source: Cells. 2021 Feb 13;10(2):385. doi: 10.3390/cells10020385 (PMC7917727; doi:10.3390/cells10020385)
Supplement: Supplementary file 1 [file cells-10-00385-s001.pdf]

Gut Microbiome Profiles and Associated Metabolic Pathways in HIV-Infected Treatment-Naïve Patients

Wellinton M. do Nascimento [1,2], Aline Machiavelli [1,2], Luiz G. E. Ferreira [3], Luisa Cruz Silveira [1], Suwellen S. D. de Azevedo [4], Gonzalo Bello [4], Daniel P. Smith [5], Melissa P. Mezzari [5], Joseph F. Petrosino [5], Rubens Tadeu Delgado Duarte [6], Carlos R. Zárate-Bladés [2,*,†] and Aguinaldo R. Pinto [1,†]

Supplementary Figures:

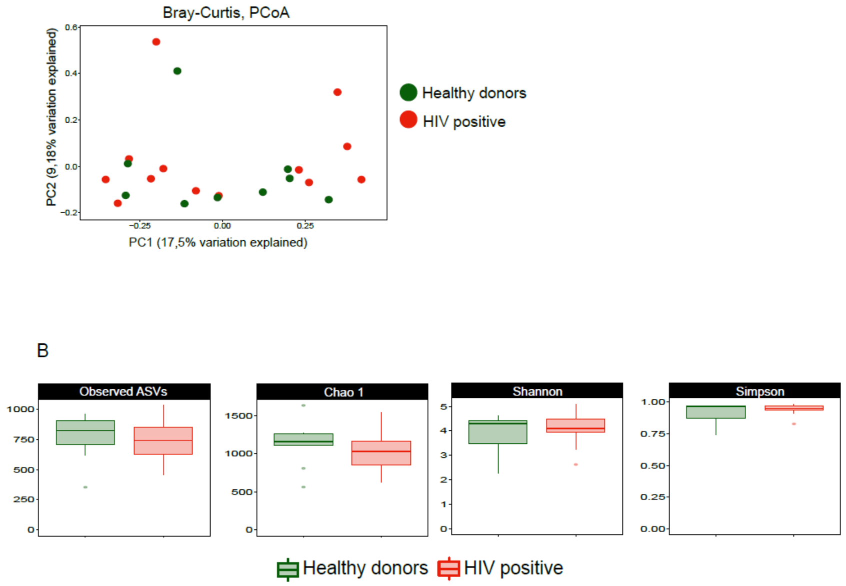

Figure 1. Comparison of gut microbiota of HIV-1 positive and healthy donors. PCoA of Bray Curtis distances among groups (a). Total number of ASVs observed, Richness (Chao1) and diversity (Shannon and Simpson) indices (b).

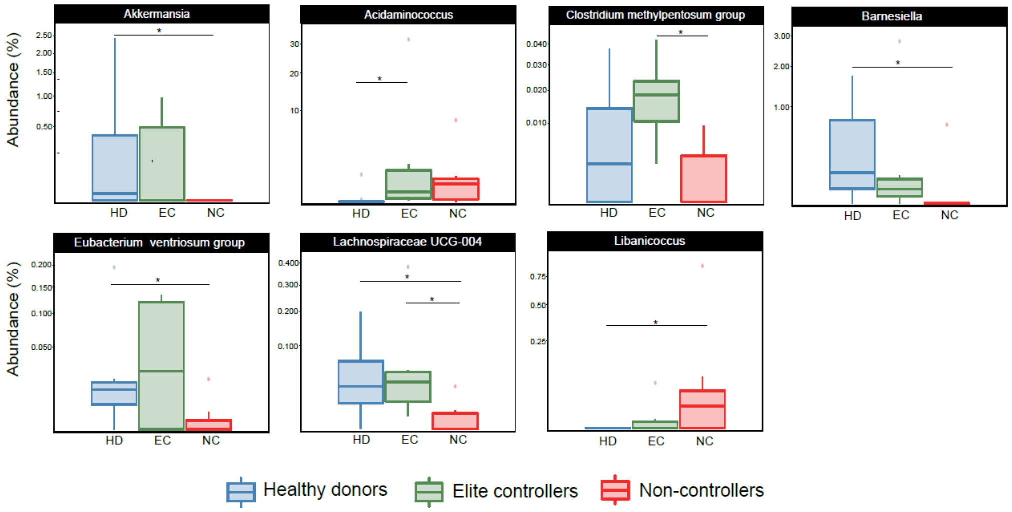

Figure S2. Comparison of gut microbiota of healthy donors (HD), elite controllers (EC) and Non-controllers (NC). The relative abundance of genera identified by LEfSe as being differentially abundant between groups were compared using the Kruskal-Wallis test. **P* values <0.05.
